# PulseNet International: Vision for the implementation of whole genome sequencing (WGS) for global food-borne disease surveillance

**DOI:** 10.2807/1560-7917.ES.2017.22.23.30544

**Published:** 2017-06-08

**Authors:** Celine Nadon, Ivo Van Walle, Peter Gerner-Smidt, Josefina Campos, Isabel Chinen, Jeniffer Concepcion-Acevedo, Brent Gilpin, Anthony M. Smith, Kai Man Kam, Enrique Perez, Eija Trees, Kristy Kubota, Johanna Takkinen, Eva Møller Nielsen, Heather Carleton, FWD-NEXT Expert Panel

**Affiliations:** 1 Public Health Agency of Canada, National Microbiology Laboratory, Canada; 2 These authors contributed equally to this work; 3 European Centre for Disease Prevention and Control (ECDC), Stockholm, Sweden; 4 Centers for Disease Control and Prevention, United States; 5 National Institute of Infectious Diseases “Dr Carlos G. Malbran”, Argentina; 6 Institute of Environmental Science and Research Limited; Christchurch, New Zealand; 7 National Institute for Communicable Diseases, South Africa; 8 Chinese University of Hong Kong, Hong Kong Special Adminstrative Region, China; 9 Pan American Health Organization/World Health Organization, Washington, DC, United States; 10 Association of Public Health Laboratories, United States; 11 Statens Serum Institut, Denmark; 12 The members of the FWD-NEXT Expert Panel are listed at the end of the article

**Keywords:** Foodborne infections, laboratory surveillance, molecular methods, public health policy

## Abstract

PulseNet International is a global network dedicated to laboratory-based surveillance for food-borne diseases. The network comprises the national and regional laboratory networks of Africa, Asia Pacific, Canada, Europe, Latin America and the Caribbean, the Middle East, and the United States. The PulseNet International vision is the standardised use of whole genome sequencing (WGS) to identify and subtype food-borne bacterial pathogens worldwide, replacing traditional methods to strengthen preparedness and response, reduce global social and economic disease burden, and save lives. To meet the needs of real-time surveillance, the PulseNet International network will standardise subtyping via WGS using whole genome multilocus sequence typing (wgMLST), which delivers sufficiently high resolution and epidemiological concordance, plus unambiguous nomenclature for the purposes of surveillance. Standardised protocols, validation studies, quality control programmes, database and nomenclature development, and training should support the implementation and decentralisation of WGS. Ideally, WGS data collected for surveillance purposes should be publicly available, in real time where possible, respecting data protection policies. WGS data are suitable for surveillance and outbreak purposes and for answering scientific questions pertaining to source attribution, antimicrobial resistance, transmission patterns, and virulence, which will further enable the protection and improvement of public health with respect to food-borne disease.

## Introduction

Almost one in 10 people in the world become ill every year due to consumption of contaminated food; diarrhoeal diseases are the most common cause of illness, with 550 million cases and 230,000 deaths every year [1]. Children under five years of age bear 40% of this burden along with potentially life-long sequelae [1]. *Campylobacter jejuni/coli* and *Salmonella enterica* are the most common causes of bacterial diarrhoea globally and are responsible for ca 96 and 80 million infections every year, respectively [1].

**Figure 1:**
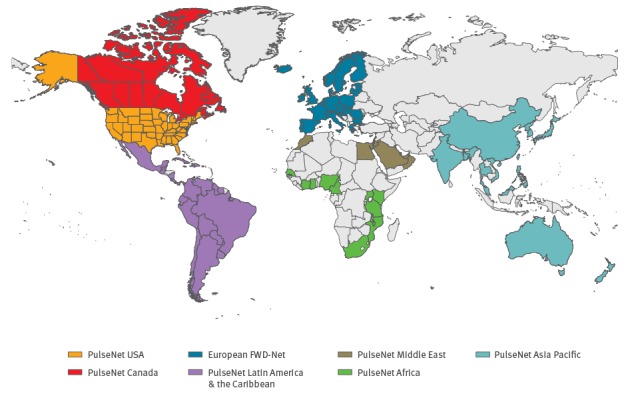
Map of PulseNet International participating countries, May 2017

FWD: food- and waterborne disease; USA: United States.

PulseNet International is a global laboratory network dedicated to bacterial food-borne disease surveillance, comprised of the national, regional and subregional laboratory networks of Africa, Asia Pacific, Canada, Europe, Latin America and the Caribbean, the Middle East, and the United States (US); 86 countries in total (Figure 1) [2].

The mission of PulseNet International is to implement standardised genotyping methods and share information in real-time within regional and national laboratory networks to support surveillance and outbreak response enabling the direct comparison of inter-laboratory data irrespective of geography.

The primary method of molecular subtyping used by PulseNet International for the identification and investigation of outbreaks has been pulsed-field gel electrophoresis (PFGE), with multilocus variable-number tandem repeat analysis (MLVA) applied to selected organisms [3]. The use of standardised, validated protocols and analysis procedures by all participants coupled with consistent interpretive criteria has enabled countless successful outbreak investigations, both within single countries and also spanning across borders [4]. Current PulseNet methods, PFGE and MLVA, are no longer considered cutting edge but have been extremely efficient in driving the detection, investigation and control of food-borne infection outbreaks in the past 20 years due to the demonstration of high typeability, reproducibility, discriminatory power, and good epidemiological concordance [5]. For timely and effective surveillance and outbreak response, data must remain comparable at all times among laboratories; any modifications on existing methods or introduction of new methods must be carefully validated and implemented by *all* network members in order to be effective and to avoid disrupting the surveillance due to backwards incompatibility issues [3,6]. Although new methods are often tested within the network they rarely make it into routine surveillance for this reason. Whole genome sequencing (WGS) has benefits that outweigh the challenges of disrupting the surveillance within the network.

WGS has shown superior sensitivity, specificity and more timely resolution to outbreak clustering compared with traditional methods [7-14]. Examples of the ability of WGS to facilitate emergency response are also demonstrated by International Food Safety Authorities Network (INFOSAN) emergency alerts; at least thirteen of the 48 biological events reported in 2014 and 2015 were supported with WGS [15]. Other strengths of WGS are its applicability to all organisms and its potential to provide multiple tests *in silico* from a single assay. These include subtyping tests, inferring biological properties (e.g. virulence genes, antibiotic resistance), and other phenotype predictions such as serotype. Moreover, genome sequencing elucidates the actual phylogenetic relationship among isolates. This renders the data useful for answering broader questions outside the relatively narrow scope of outbreak detection and response. For example, the same data used for routine surveillance could also be used for precise microbiological attribution studies, to elucidate transmission pathways and common properties of persistent strains, and to identify potential intervention points along the food safety continuum.

The vision of PulseNet International is for WGS to be used in all public health laboratories to identify, characterise and subtype food-borne pathogens, largely replacing existing phenotypic and molecular methods in support of preparedness and response to food-borne illness at the local, national, regional and global levels. This paper provides considerations of the critical technical and practical aspects of WGS from the perspective of standardised international laboratory-based surveillance and the prerequisites for routine implementation in public health.

## Whole genome sequence generation

### Preparation and sequencing

There are several technologies available for genome sequencing. Collectively known as massively parallel sequencing, they produce billions of nt sequences during each run, where each genome is sequenced multiple times in small random pieces (reads and contigs) to generate very large datasets [16]. Even though sequencing platforms (e.g. Illumina, etc.) have different biochemistry and arrays, the workflow is similar: (i) DNA extraction; (ii) library preparation, which usually includes shearing the DNA either mechanically or enzymatically, adding adaptors and barcodes/indexes, and amplification; (iii) template preparation, either by bridge amplification or emulsion PCR; and (iv) sequencing (the sequencing run itself is highly automated). 

### Data processing

Processing data from WGS, regardless of the platform used, follows the same general workflow. Fragments or ‘reads’ (FASTQ format) from sequencing runs are trimmed to remove adaptor and barcode sequences (added during library generation), and low-quality reads. Depending on the analysis method chosen, the reads may or may not need to be assembled. Assembling is the process of placing all of the reads together in the correct order to create a small number of contiguous sequences, known as contigs (FASTA format). This can be done using a known reference sequence (closed genome) to guide the assembly, or it can be done de novo (draft genome), i.e. without prior knowledge of the expected order. Especially the latter is computationally relatively demanding for the four main species involved in food-borne diseases: *Salmonella* spp, *Campylobacter* spp, *Listeria monocytogenes* and Shiga toxin-producing *Escherichia coli* [17]. With current technologies, due to the short length of the individual reads compared with repetitive regions, a genome for these species is also rarely fully assembled (closed genome).

## Analysis methods

### K-mer, SNP, and gene-by-gene methods for *in silico* subtyping

There are many ways to analyse whole genome sequence data, including methods that will replace traditional molecular subtyping methods. Three common approaches for this are k-mers, single nucleotide polymorphisms (SNP), and the gene-by-gene based – i.e. extended multilocus sequence typing (MLST) based on WGS – approach. These methods have good epidemiological concordance, but differ in other features such as amenability to standardisation, stable nomenclature, scalability, the need for reference strains or assemblies, and computing and bioinformatics resource requirements (Table 1).

**Table 1:** Key features of k-mer, single nucleotide polymorphism (SNP) and multilocus sequence typing (MLST) approaches relevant to laboratories providing routine public health functions

Features	K-mer	SNP	MLST
Epidemiological concordance	Intermediate	High	High
Discrimination	Intermediate	High	High
Stable strain nomenclature	No	No	Yes
International standardisation	No	No	Yes
Scalability	No	No	Yes
Speed	Intermediate	Slow SNP calling, slow comparisons	Slow allele calling, fast comparisons
Local computing requirements	Low	High	Low
Local bioinformatics expertise required	Yes	Yes	No
Curation of database	No	No	Yes

SNP: single nucleotide polymorphisms; MLST: extended multilocus sequence typing.

Briefly, k-mers have lower discriminatory capacity than the two other methods and are mainly useful for crude and rapid initial sequence comparison of isolates where maximum resolution is not needed and as a tool for detection of species [18]. The SNP approach is highly discriminatory but sensitive to the selection of the reference the SNPs are called against; this means that if two laboratories use a different reference for their SNP calling of the same outbreak the SNP differences of isolates not included in the analysis in both laboratories cannot be directly compared [18]. SNP analysis is computationally intensive and might therefore be rather slow; access to high performance computing is mandatory for analysing large sequence sets. Additionally, many SNP pipelines are currently command-line based, requiring substantial bioinformatics knowledge by the end-user. This is likely to change as the field progresses and pipelines continue to evolve to more user-friendly, ‘plug and play’ formats. The method chosen for standardised surveillance by PulseNet International is the gene-by-gene approach. 

### Whole genome MLST, the method chosen by PulseNet International 

For integration into routine public health surveillance and for maintaining inter-laboratory comparability, the gene-by-gene, i.e. extended MLST, approach offers a number of compelling features. The extended MLST schemes assess information from coding regions only, and collapse different types of mutations into a single allelic change. Allele information is assessed by comparing new sequences with an allele database that contains all genes (‘loci’) present in the typical several hundred strains used to create the scheme. The number of genes assessed may range from typically seven housekeeping genes to a several thousand [19]. The biggest schemes contain the genes in the core genome (genes present in nearly all strains of the same species, core genome (cg)MLST), or the whole or pan genome (all core genes plus accessory genes present in any strain used to create the allele database, whole genome (wg)MLST). Custom sets of any number of genes may also be analysed. Constructing and validating a reliable cg- or wgMLST scheme that is also accessible to all and simple to run is a significant undertaking but once implemented such schemes could be easy to work with by the end-users (microbiologists and epidemiologists). Nevertheless, the output from wgMLST analyses may also need consultation with experts to ensure proper interpretation. The allele calling process can be automated but is fairly slow; once alleles are called the analytical process is fast. In contrast to SNP analysis, wgMLST comparisons are much faster and may run on ordinary desktop computers, as an allelic profile is simply a string of integers.

Extended MLST schemes are phylogenetically relevant and at least in their most extensive form, wgMLST, they appear to be as discriminatory as SNP comparisons for *Salmonella* and *Listeria*, and provide significantly improved resolution and epidemiological concordance compared with molecular methods (data not shown).

Backwards compatibility of WGS with the existing gold standard methods PFGE and MLVA is very limited, regardless of the WGS analysis method. The short reads are not amenable to a complete assembly, which would be required to predict a PFGE restriction pattern, and in particular do not assemble the repetitive regions assessed by MLVA correctly. To mitigate the effects of losing comparability, some laboratories are choosing to perform both molecular and WGS-based subtyping in parallel for a period of time. The seven-gene MLST pattern can readily be extracted from WGS data.

The technical performance of wgMLST along with its scalability and amenability to standardisation and stable nomenclature (see section below) plus the computational and bioinformatics prerequisites realistic for many public health laboratories position wgMLST as the method of choice at this time for PulseNet International.

## Implications for information technology (IT) infrastructure and bioinformatics expertise

WGS presents a number of challenges to the IT infrastructure of most PulseNet laboratories, which often operate in tightly regulated Windows-operating system-based computing environments and of which some may not always have stable power supply or Internet connections. Key issues for implementing WGS are bioinformatics expertise and software, and especially for larger laboratories storage space and computing power.

### Storage space

Each sequencing run generates gigabytes (GB) of data, with sequence read sets (SRS) of 100–500 megabyte (MB) in size for each isolate. Many laboratories anticipate analysing thousands of isolates each year, and storage on existing systems will likely be prohibitively expensive. Generally speaking, it is desirable to retain the SRSs (and not just the final processed data assembled) so that they may be used for future analyses by using alternative methods such as SNPs or assessment of mobile genetic elements e.g. from phages. Some PulseNet International members currently store the SRSs within their own reference laboratory’s data storage. Some also submit them in close to real-time to one of the databases of the International Nt Sequence Database Collaboration (INSDC, http://www.insdc.org), such as the Sequence Read Archive (SRA) at the National Center for Biotechnology Information and the European Nt Archive at the European Molecular Biology Laboratory, as well as the DNA Databank of Japan Sequence Read Archive (DRA). Using a public domain archive removes the storage cost from individual laboratories and makes the data available to the wider scientific community in perpetuity, but also necessitates public data sharing. This is problematic for some countries with respect to data protection (see section on data sharing below). Additionally, it is expected that very few countries in the future will have the capacity to store the ever growing amount of raw sequence data in-house. Strategies to manage the information associated with each isolate (i.e. the descriptive data, sometimes referred to as meta-data) are being developed and implemented by some PulseNet International network laboratories (see data sharing section below). Another potential storage solution consists of commercial clouds (e.g. BaseSpace, Amazon S3), with users paying per GB of storage; storage costs using these options are typically lower than in-house solutions; however, many organisations prohibit the use of commercial clouds due to institutional data security policies. Additionally, if an outside-house storage solution is pursued, Internet connections with adequate bandwidth must be available. Given the great diversity in resources across PulseNet International laboratories, a one-size-fits-all solution might not be possible but renders publicly available storage options compelling.

### Computing power, bioinformatics expertise, and software

Laboratories have three options for bioinformatics tools: in-house pipelines, web and/or cloud-based tools, and outsourcing; each has pros and cons. Many in-house bioinformatics pipelines utilise Linux-based tools, which while powerful, cannot be easily run on Windows- operating system-based computers and are frequently not supported by institutions’ information technology (IT) departments. 

**Figure 2:**
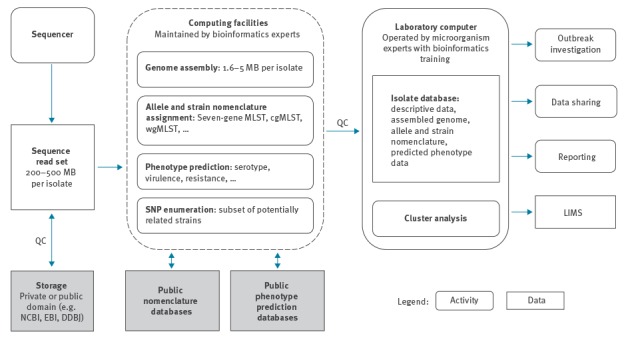
Potential solutions for computing and storage in PulseNet International laboratories, May 2017

Cloud-based and publicly available computing and bioinformatics may provide a solution for analysis (e.g. the Center for Genomic Epidemiology, http://www.genomicepidemiology.org/). However, public health laboratories operate under strict quality assurance and accreditation requirements, necessitating guaranteed access to computing and bioinformatics tools. Within a single country or region, cloud-based bioinformatics tools can be maintained by local specialists, be partitioned from secure corporate or in-house computing networks as needed, and in many cases be scaled to meet demand (Figure 2). For PulseNet International laboratories with modest numbers of isolates, making use of cloud-based or other web-based public analysis tools may facilitate entry into WGS (as could outsourcing the actual sequencing instead of purchasing the equipment). The actual analysis tools can be used from open sources or commercial software.

Cg: core genome; DDBJ: DNA Data Bank of Japan; EBI: European Bioinformatics Institute; LIMS: Laboratory Information Management System; MB: megabyte; MLST: multilocus sequence typing; NCBI: National Center for Biotechnology Information; QC: quality control; SNP: single nucleotide polymorphism; wg: whole genome.

This workflow separates storage of the sequence data, from the intensive bioinformatics analyses that need to be performed on them, in particular genome assembly and allele calling. This allows performing these analyses on a dedicated computing cluster either in-house or in the cloud, where they can be maintained and updated by bioinformatics experts who may not reside within the same organisation or even country as the PulseNet laboratory. No confidential information is handled on this computing cluster. Within the PulseNet laboratory, microorganism experts will perform quality controls, cluster analysis on MLST data, as well as evaluating virulence and other factors. Following data interpretation, laboratory experts will use this information for outbreak investigations, reporting and sharing with other agencies. When required, SNP analysis will be performed on subsets of closely related strains.

Additionally, outsourcing analyses to a third party is an alternative, e.g. a PulseNet regional coordinator may serve as a third party analyst as it was done in PulseNet Latin America and the Caribbean for the analysis of PFGE data while waiting for individual participants to acquire the analytical software [2,20].

Due to the nature of public health and regulatory decision making action in food safety, robustness is essential as subtyping results often provide the critical evidence during food recalls, outbreak investigations, and prosecutions; they must be defendable in a court of law. For this reason, the ability to track the versions of software tools and parameters is a basic requirement, i.e. the ability to precisely recreate analyses from the past and arrive at the same results is a necessity. Presently, many PulseNet International laboratories do not have ready access to bioinformatics expertise. In deciding to use open source or commercial software, issues to consider include the need and ability to modify and tailor the source code, the ability of the non-bioinformatician PulseNet participants to learn and use the tools, the process for and cost of maintenance and updates, version control and other quality measures, and the cost of licenses and bioinformatics personnel. The ability to store and analyse associated descriptive and other laboratory data alongside the sequence data also needs to be considered.

## Nomenclature

Despite achieving success in standardising molecular subtyping and inter-laboratory comparability across 86 countries, PulseNet International has not been able to implement global PFGE nomenclature for food-borne pathogens, largely due to the complexities (and costs) of implementing a central, global PFGE database requiring frequent manual curation. To date, each country or region maintains their own PFGE databases and nomenclature, with the exchange of data files to compare results across disparate nomenclatures as needed. In addition to its superior performance, WGS provides at long last the opportunity for a truly global nomenclature, facilitating laboratory-based surveillance at the international level and opening the door to future reductions in the burden of food-borne disease. Gene-by-gene-based approaches to WGS-based subtyping are highly suitable to stable nomenclature and, with international agreement to use the same scheme, provide a natural choice for worldwide implementation via PulseNet (see previous section). A detailed consideration of nomenclature is provided by the European food- and waterborne disease (FWD)-NEXT Expert Group [21].

### Allele nomenclature

**Figure 3:**
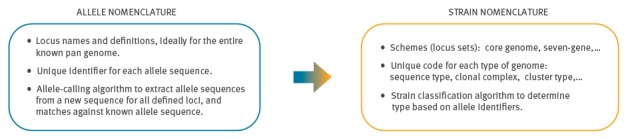
Allele and strain nomenclature

Allele nomenclature provides the names and definitions of all loci included in the wg/cgMLST scheme, as well as correspondence between allele sequences and allele identifiers (Figure 3).

The latter consists of unique identifiers, integers, that each correspond to a particular allele sequence for a pre-defined locus on the genome, and which do not change over time. Assignment of allele identifiers converts the WGS data for an isolate into a series of identifiers, one per locus. The resulting allelic profiles are used to determine genetic similarity between isolates. For PulseNet International laboratories to maintain real-time outbreak detection and inter-laboratory comparability, it is crucial that allele calling can be done reliably in real-time; i.e. automated, and that the nomenclature scheme used is the same in all laboratories. This will ultimately require a global allele-calling algorithm, which is still in the early stages. Issues to be addressed are the use of sequence reads vs assemblies, assembly method, locus start and end definitions, parameters for locus identification and allele sequence extraction, algorithm accuracy per locus, etc.; the amount of manual curation may initially be high but ultimately the actual allele calling will be fully automatic. The wg/cgMLST approach will also necessitate an international database of alleles; there are several features of such a database that would be highly beneficial to international public health users (Table 2).

**Table 2:** General requirements for a global allele nomenclature database, May 2017

ID	Description
1	Submission of sequence data and any subsequent allele calling can only be done by and for registered users. The nomenclature database content on the other hand, including the full set of known unique allele sequences and their identifiers, must be publicly accessible.
2	All nomenclature related functionality of the database must be free of charge to end users. Long-term sustainability and portability to other servers must be addressed.
3	The database must have close to 100% guaranteed uptime and have sufficient bandwidth to support upload of data by organisations worldwide. Sufficient computing power must be available to perform quality controls and allele calling in real time.
4	It must be possible to submit either raw reads or individual allele sequences, in order to retrieve the corresponding allele identifiers as well as any quality control results. Raw reads or any derived data other than individual new alleles may not be stored permanently in the database or used for any other purpose than deriving allele nomenclature. If needed for practical reasons, submission of raw reads can be implemented at a later stage.
5	There must be an open interface for machine-to-machine communication that covers all of the publicly available functionality. A formal process to incorporate input and agreement from stakeholders on changes to the system must be in place.
6	It must be possible for authorised curator users to annotate individual allele sequences with information, e.g. to include them as a reference allele, and to add additional loci to derive allele nomenclature for, as the known pan genome grows.

Organisations producing WGS data have differing policies on data sharing, so a global allele nomenclature database that strives to meet the needs of most is optimal. For example, it must be possible to submit not only whole genomes as raw reads or assembly, but also individual new alleles detected through the use of a local cached allele nomenclature database. In case of full genomes, it must be possible to have them automatically deleted once alleles have been called unless the submitter wants the full sequence to become available to the general public and/or the allele database curators. Because of the substantial size of raw read data, an allele calling platform would also need sufficient bandwidth to support such large amounts of data being continuously uploaded by organisations worldwide, along with computational resources available to process alleles and return results in real-time. A flexible system that has an open interface would facilitate integration with existing organisations’ or other third party systems. Quality control must be carefully planned as part of allele calling, as poor quality or dubious alleles, or alleles that match several paralogous genes, should not be used as reference alleles due to the reduction in reproducibility and deterioration of quality. For all submissions, the provision to the user of an automated quality assessment is essential. The selection of additional reference alleles will require manual intervention by curators, but this is expected to be infrequent. New loci should not routinely be added to an existing wgMLST scheme since this invariably will also require revision of the strain nomenclature database (see next section). Security features would mitigate inadvertent or malicious use (e.g. submissions of large amounts of artificial sequences) while ensuring that individual allele sequences and their identifiers remain publicly accessible – perhaps by requiring user registration for submissions. Finally, a process to solicit input from stakeholders and the development of a collaboration agreement or terms of service would facilitate use by many public health organisations. Until the global allele nomenclature database has been established, PulseNet International members have informally agreed to work with local or regional versions of the same extended MLST scheme and use internally the same allele calling method for each pathogen.

### Strain nomenclature

Strain nomenclature is a construct devised to classify and accordingly label an isolate, placing it into a designated category within the diversity of the species. It is essential in food-borne disease outbreak investigations for simplified and rapid communication among stakeholders. These include epidemiologists, medical and food safety officers, inspectors, and healthcare and environmental health professionals, wherein rapid and effective communication is best achieved through simple tables, line lists, outbreak reports, and person-to-person conversations, without the need to explain the topology of a phylogenetic tree. A strain nomenclature derived from allele identifiers but also reflecting phylogeny (and epidemiologically relevant) would be ideal. Such nomenclatures are hierarchical when they consist of codes that contain several levels of similarity [21,22]. When two isolates share only part of the code – e.g. ‘1.2.3’ vs ‘1.2.4’ – they are similar to some extent since they are both classified as ‘1.2’ and more similar than isolates that differ in the second digit, e.g. ‘1.2.3’ vs ‘1.4.9’, which in turn are more similar than isolates that do not have the first digit in common, e.g. ‘1.2.3’ vs ‘2.1.4’. The definition of a species’ core genome, i.e. the loci common to nearly all isolates of a particular species (or lineage) is critical for the development of strain nomenclature. For some pathogens, whole genome or pangenome, i.e. all loci detected in any isolate of a species, may also be used for strain nomenclature. Core genomes have been suggested for *L. monocytogenes*, *C. jejuni/coli, S. enterica,* and *E. coli* (http://bigsdb.pasteur.fr/listeria/listeria.html;http://pubmlst.org/campylobacter; https://enterobase.warwick.ac.uk) [23,24]. The stability and accuracy of taxonomical nomenclature is not yet well established, although they are expected to be suitable for surveillance purposes. For those pathogens for which a hierarchical nomenclature may not be possible, categorical nomenclatures akin to the sequence types and clonal complexes for seven-gene MLST can always be derived. The algorithm for strain classification should ideally be stable, i.e. reproducibly return the same code for the same isolate on repeated testing. As is the case for the allele nomenclature database, an open interface for machine-to-machine communication and authentication of users should also be in place. It has much lower infrastructure requirements than allele nomenclature since only allele identifiers need to be submitted. Global strain nomenclature schemes should ideally be curated by the subject-matter experts who developed the underlying MLST schemes; this will require collaboration and cooperation between public health authorities and academic experts.

## Data sharing

Rapid sharing of subtyping data between country or regional coordinators has been achieved in PulseNet International to-date by the exchange of PFGE data files on a members-only Internet-based discussion board on an as-needed basis. Typically, this occurs after a potential outbreak has already been flagged by one country/region, with the exception of the US and Canada who permit direct access to each other’s national databases. National or regional databases are accessed directly by internal network members. With the limited utility of PFGE data outside of outbreak detection and response, there has not been cause to make these data more widely available on a routine basis. Also, sharing of real-time PFGE results outside the network has historically been strictly controlled, executed according to agreements negotiated by coordinating country/regional laboratories and their submitters (typically state, provincial, or local laboratories) whose individual privacy legislations stipulate conditions for sharing. 

However, genome sequences can be used for many more purposes than routine public health surveillance and outbreak response, in particular they are highly suitable for basic research and activities aligned with the One Health approach [25]. Ideally, the genome sequences collected for surveillance purposes should be publicly available, if not in real time then within a reasonable time frame, e.g. within 12 months of sequence generation. These data should comprise both the sequences and some descriptive data about the isolate, as the sequences themselves alone have extremely limited utility. Publicly available repositories within the INSDC accessible via the Internet would be the ideal storage location, i.e. the SRA, the European Nt Archive and the DNA Data Bank of Japan as these are synchronised with each other on a regular basis. Each organisation or country/region determines itself which sequence data to upload, along with what descriptive data (e.g. time, place and type of isolate) would be uploaded to these repositories. Current data sharing procedures in some PulseNet International participating laboratories show promise in providing a suitable balance between protecting the privacy of patients and the utility of data generated and shared in real-time for global surveillance; these procedures include providing very little descriptive data for each isolate initially followed by the addition of information about the isolate after a 6–12 month delay. 

At present, not all public sequence repositories allow for user-defined controls (e.g. sharing WGS data with a specific user subset immediately after upload and during a period of embargo before public release); however, such functionality as well as an improved interface may accelerate implementation of WGS and data sharing by more organisations. Given the expertise, infrastructure, mission and stability of the public repositories, integrating the global allele nomenclature databases, the allele-calling algorithms and possibly the strain nomenclature databases into these public spheres with an appropriate curator interface for subject matter experts would be desirable at the global level. Storing the allele identifiers along with the WGS data in the sequence repository would effectively make the latter searchable for the presence of loci, specific alleles and specific mutations. Since the principle of allele nomenclature is species independent, it could even be applied to all species rather than only those relevant for food- and waterborne diseases, to the benefit of all users of these databases.

## Validation

Like other subtyping methods, WGS-based tests must be thoroughly validated before implementation in routine public health practice. For a global network like PulseNet International, validation ensures that the method is applicable for strains encountered by all network members, performance is suitable, and it also generates interpretive guidelines necessary for the consistent interpretation of WGS results. Methods are subject to three phases of validation: internal, external (see below for explanation), and post-implementation evaluation and refinement. Ideally, all phases should be completed before implementation network-wide. However, the rapid demonstration of the superior technical performance of WGS compared with methods previously accepted as gold standards (e.g. PFGE, MLVA) has accelerated its implementation in many laboratories, before the formal completion of validation at the international level. Regardless of current individual country status, validation is ultimately necessary to ensure evidence standards for public health decision making can be consistently met.

### Internal validation

The purpose of internal validation is to verify the robustness and technical performance of the method using a well-defined set of isolates. For internal validation of wg/cgMLST-based approaches, for example, this includes an assessment of each locus plus the scheme as a whole for reproducibility, stability, discrimination and epidemiological concordance, and a comparison with current gold standards.

### External validation and post-implementation evaluation

During external validation, the portability and inter-laboratory robustness of the method are tested on a common isolate set in a wider number of laboratories. Typically this includes geographically dispersed laboratories with different levels of expertise and access to a wide variety of equipment and reagents. The external validation should also include prospective (or retrospective, if necessary) testing of isolate sets collected from each laboratory’s routine operations. Once implemented, periodic evaluations are conducted for continual improvement. This is done to detect problems not identified during the initial validation, to assess the impact of any proposed changes to laboratory equipment and reagents, and to accommodate new bioinformatics methods. With the fields of genomics and bioinformatics currently experiencing rapid rates of advances, international systems such as PulseNet should be flexible enough to allow improvements to the process while protecting the integrity of inter-laboratory comparability and quality control.

## Quality control

While quality control for individual laboratories is important, it is critical for a network wherein the results generated by laboratories other than your own are relied upon to support local and multi-jurisdictional public health decision making. A rigorous quality control programme ensures that correct results are reliably produced by all participants at all times and provides checkpoints during the process to flag and remove potentially incorrect results.

### Routine quality parameters

Quality of the raw reads must be assessed; if the minimum quality metrics are not met, the reads must be discarded and sequencing repeated. All platforms produce raw reads of both sequences and the individual quality scores. A primary quality metric is coverage, defined as the number of raw reads for the average read length over the genome (i.e. the number of times each base in the genome is contained in individual raw reads). A minimum coverage of 30x is typically enough for routine surveillance, but this is platform-dependent and also may vary by organism [21,26]. Other quality metrics to be checked routinely include the average Phred or Q scores as a measure of base-calling accuracy. Low quality reads should not be uploaded to a repository or PulseNet database, open or closed to the public. Taxonomy check should also be included in the basic raw read quality check since isolate mix-ups and mixed cultures are the most common errors encountered in the laboratories generating sequencing data. Similarly, routine quality parameters such as the proportion of core genome loci detected through allele calling should also be implemented for the bioinformatics pipeline(s) used for analysis.

### Ensuring standardisation and competence

As basic tenets of quality control, written standard operating procedures must be in place for both sequencing and analysis and the procedures should only be performed by trained personnel. Participation in an external quality assessment (EQA) programme is recommended. While each country/region sets its own quality control requirements, many PulseNet International participating countries submit and pass an organism-specific certification test before they are permitted to submit data to a repository or to most PulseNet country/regional databases (although this is not the case in Europe or PulseNet Asia-Pacific). The certification process documents each participant’s highest level of competence in producing raw sequence data, performing analysis using bioinformatics tools, and checking data quality (participants may be certified in producing raw sequence data, analysing the results, or both). Initial PulseNet certification is followed by annual proficiency tests to ensure that competence is maintained. These quality control procedures are largely based on the well-established PulseNet International quality control programme for molecular subtyping; however, they may be further modified as needed to best suit WGS, as part of the continual evaluation and refinement process.

## Implementation

### Global whole genome sequencing implementation is not all-or-nothing

The benefits of genome sequencing have driven its use for public health decision making despite the lack of fully developed systems and infrastructure [27]. Across members of PulseNet International, the maturity and complexity of internal country/regional laboratory surveillance networks, the availability of funding and human resources, as well as the relative importance of infections caused by unsafe food among all infectious diseases differ across countries, according to each country’s own priorities and resources. Thus, the strategy and timing for implementation of WGS for food-borne disease surveillance will vary around the world. At the same time, the costs of WGS are often already lower than the currently used characterisation methods including PFGE and MLVA and will likely decrease further [21]. Recently, recommendations have been published to guide both developed and developing countries in determining their readiness for implementing WGS [28]. With readiness dependent on a wide variety of political, technical, economic and political factors; e.g. infrastructure, equipment, training, operating funds, policies, etc.; each country will set their own timeline for implementation. Some countries have been able to make rapid transitions to WGS (i.e. within 12 months or less), others have had a slower pace spanning multiple years. Internationally, a gradual transition from traditional molecular methods to genome sequencing would ensure that countries not able to immediately implement WGS are not separated from the rest of the public health community [21]. Waiting for universal readiness before moving forward is also not advised; this would delay the manifestation of WGS benefits to food-borne disease management and prevention overall. There is a risk of dividing countries according to genomics capacity, furthering health inequalities due to economic status and hampering efforts to reduce disease burden and further efforts towards One Health [25].

### The role of PulseNet International’s public health laboratory network

PulseNet International’s extensive network of laboratories, and history of standardisation and sharing, uniquely position it to guide WGS implementation worldwide in a manner that minimises isolating individual countries or regions. As revolutionary as genomics is for food-borne disease (and virtually all areas of infectious disease public health), these challenges of international compatibility are not new. These very same issues were faced during the implementation of PFGE in PulseNet International starting two decades ago [2]. Global standardisation was achieved through the network’s capacity building and training activities, consensus decision making among participants, and a careful balance between scientific merit and practical issues, these strengths provide the foundation to build a new system based on WGS. The members of PulseNet International are almost exclusively focused on the needs of routine surveillance for outbreak detection and response, which enables advances in genomics and bioinformatics to be harnessed and tailored to those specific needs while simultaneously advocating for the availability of data for broader purposes. The sharing of experiences and ‘lessons learned’ is another key role of PulseNet International; routine communication and sharing of protocols, policies, implementation plans, etc., across the network enables all members to benefit from the experience of early adopters. 

Other national and international initiatives and networks lead or contribute substantially to the development of genomics and bioinformatics in the area of food safety. For example, the GenomeTrakr network (US Food and Drug Administration) has pioneered open source tools, public data sharing, and the application of WGS to regulatory and compliance activities [29]. PulseNet International is the public health-focused network of laboratories responsible for surveillance and outbreak response, which is complementary to GenomeTrakr activities. In the European Union, the European Centre for Disease Prevention and Control and the European Food Safety Authority collaborate to collect and analyse molecular typing data in a single database, providing centralised surveillance capability. The Global Microbial Identifier initiative is the forerunner for planning global genomics systems for all infectious diseases in all sectors of science and beyond [7]. The European COMPARE project leverages INSDC infrastructure and is working to provide a platform to support WGS data sharing and analysis (http://www.compare-europe.eu/). Additional organisations also play pivotal roles in international food safety, including the World Health Organization of the United Nations’ INFOSAN and Global Food-borne Infections Network, and the Food and Agricultural Organisation of the United Nations. These organisations are also leaders in capacity building and knowledge transfer for WGS and its impacts on food-borne disease surveillance, as well as for regulatory and policy framework guidance.

### Beyond the laboratory

For the integration of WGS for routine surveillance, all public health-related professionals must be prepared for the use of genomic data to support public health decision making. WGS should not be portrayed as a panacea; it does not replace the need for high quality epidemiological data to be interpreted in context with laboratory results [30]. Knowledge transfer must occur not only for the participating public health laboratories, but also for the national and regional epidemiologists and other immediate stakeholders. PulseNet member laboratories often serve as the interface between food-borne disease laboratory and epidemiology, and between public health and food monitoring. Understanding basic genomics and bioinformatics concepts by all members of the food safety continuum is needed.

## Conclusion

To meet the needs of real-time surveillance, PulseNet International will standardise WGS-based subtyping using extended MLST-based approaches; specifically, wgMLST. This delivers optimal resolution and epidemiological concordance while providing unambiguous nomenclature. In addition, it is computationally efficient and realistic for most public health laboratories to use on a daily basis. Standardised protocols, validation studies, quality control programmes, database and scheme development, and training materials all must support the implementation and decentralisation of any new technique. As was done previously with PFGE, PulseNet International is presently disseminating these important elements among network members. Training, in particular, is a keystone for the success of standardised PulseNet activities; this will require dedicated and sustainable resources. A recent economic evaluation of PulseNet activities suggests that in the US alone, PulseNet prevents at least 270,000 illnesses from *S. enterica*, *E. coli*, and *L. monocytogenes* and saves 500 million US dollars (447 million euros) every year in medical costs and lost productivity [31]. The economic return on investment in PulseNet is approximately 70 US dollars/euros of benefit for every 1 dollar/euro invested by public health agencies. This demonstrates a significant economic and public health benefit from the system, and these impacts are expected to be even greater with the superior performance of WGS.

In order to truly standardise food-borne disease subtyping across the world, a public WGS-based nomenclature, curated where necessary, must be available as WGS data are not only suitable for surveillance and outbreak purposes, but also for answering other scientific questions (e.g. source attribution, antimicrobial resistance, transmission patterns, population structure, etc.). The WGS data themselves (including a minimum set of descriptive data) should ideally also be publicly available. To fully realise this vision, technical and political challenges must be overcome. PulseNet International will leverage its experience to guide the implementation of genome sequencing in a manner that both meets immediate public health needs and supports broader efforts to preventing and mitigating the effects of food-borne disease worldwide.

## Notes

**FWD-NEXT Expert Panel Members:** Ariane Pietzka (Österreichische Agentur für Gesundheit und Ernährungssicherheit, Graz, Austria); Eva Trost (Robert Koch-Institut, Wernigerode, Germany); Kristoffer Kiil (Statens Serum Institut, Copenhagen, Denmark); Ivelina Damjanova (Országos Epidemiológia Központ, Budapest, Hungary); Valeria Michelacci (Istituto Superiore di Sanità, Rome, Italy); Joël Mossong (Laboratoire National de Santé, Luxembourg); Eelco Franz (Rijksinstituut voor Volksgezondheid en Milieu, Bilthoven, Netherlands); Wilfrid van Pelt (Rijksinstituut voor Volksgezondheid en Milieu, Bilthoven, Netherlands); Tomasz Wołkowicz (National Institute of Public Health – National Institute of Hygiene, Warsaw, Poland); Vítor Borges (Instituto Nacional de Saúde Doutor Ricardo Jorge, Lisbon, Portugal); Cecilia Jernberg (Folkhälsomyndigheten, Stockholm, Sweden); Erik Alm (Folkhälsomyndigheten, Stockholm, Sweden); Ian Fisher (Public Health England, London, United Kingdom); Kathie Grant (Public Health England, London, United Kingdom); Tim Dallman (Public Health England, London, United Kingdom); Joakim Ågren (Statens veterinärmedicinska anstalt, Uppsala, Sweden); Martin Maiden (University of Oxford, Oxford, United Kingdom); Sylvain Brisse (Institut Pasteur, Paris, France); João André Carriço (Universidade de Lisboa, Lisbon, Portugal); Guy Cochrane (European Molecular Biology Laboratory, European Bioinformatics Institute, Hinxton, United Kingdom); Beatriz Guerra (European Food Safety Authority, Parma, Italy); Maria Teresa Da Silva Felicio (European Food Safety Authority, Parma, Italy); Daniel Palm (European Centre for Disease Prevention and Control); Joana Revez (European Centre for Disease prevention and Control); Marc Struelens (European Centre for Disease Prevention and Control).
